# Favorable outcome of pregnancy on antiretroviral treatment in a patient with HIV-infection

**DOI:** 10.1186/1471-2334-14-S4-P46

**Published:** 2014-05-29

**Authors:** Iosif Marincu, Emilian Popovici, Sorina Laitin, Valeria Bică, Patricia Poptelecan, Livius Ţîrnea

**Affiliations:** 1 Department of Infectious Diseases, Pneumology and Parasitology, Dr. Victor Babeş University of Medicine and Pharmacy, Timişoara, Romania

## 

Antiretroviral (ARV) therapy in pregnant women is particularly important for both patients and for her future child. Objectives: To present clinical and biological evolution of pregnant patients under ARV therapy combination Combivir+Kaletra (CBV+KAL) with good adherence to treatment.

The authors present the case of a patient, 22 years old, from urban area, monitored in Infectious Diseases Clinic Timişoara in 2010 with HIV infection stage C2. Personal pathologic history: oropharyngeal candidiasis, toxoplasmosis, suppurative otitis, bilateral coxofemoral and knee arthritis, HIV encephalopathy depressive disorder, hypotrophy weight, static encephalopathy, dyslipidemia, chronic HBV hepatitis. Biochemistry, bacteriology and immunology were performed in the hospital laboratory using an automatic biochemical analyzer (Konelab 301) and an automated system VITEK 2 Compact. Assessment of viral load by PCR was performed in the Laboratory of the National Institute for Infectious Diseases “Prof. Dr. Matei Balş”, Bucharest. Throughout pregnancy the patient received ARV treatment with CBV+KAL.

Results: the beginning of pregnancy: Hb=13.1 g%, H=4,270,000/cmm, Tr=285,000/cmm, L=6,000/cmm, ESR=32 mm/1h, fibrinogen=3.6 mg%, ALT=42 U/L, AST=31 U/L, cholesterol=200 mg%, triglyceride=156 mg%, glycemia=102 mg%, ASLO=200 U/L, C-reactive protein=5 mg/L, calcemia=18 mg/dL, glossal exudate and culture on Sabouraud medium=negative, sterile urine culture, positive HBsAg, CD4=361 cells/µL, VL=672 copies/mL. Gynecological examination: pregnancy 8 weeks in evolution. Counseling was pregnant and gave very good compliance and adherence to treatment. In February 2012 scheduled cesarean delivery: girl of 2800 g which until now (has 2 years) is clinically healthy, HIV negative. Father of daughter is in our records since 2000 with HIV-infection, under ARV therapy, combivir+nevirapine (CBV+NVP) with very good adherence to treatment, CD4=658 cells/µL, VL=undetectable from 2009 to the present. There were no reported side effects of ARV therapy administered (CBV+KAL) and immunological tests showed a clear improvement (CD4=420 cells/µL, VL=220 copies/mL).

Combination ARV therapy (CBV+KAL) associated with good compliance and adherence to treatment in pregnant women ensures optimal control of immunity.

